# Re-Analysis on GRCh38 with the MANE Select Transcript Resolves Previously Undetected Biallelic *ODAD4* Variants in Primary Ciliary Dyskinesia

**DOI:** 10.3390/cells15201893

**Published:** 2026-10-17

**Authors:** Petra Pennekamp, Marina Praprotnik, Bernd Dworniczak, Heymut Omran, Rim Hjeij

**Affiliations:** 1 Department of General Pediatrics, University Hospital Münster, 48149 Münster, Germany; petra.pennekamp@ukmuenster.de (P.P.); bernd.dworniczak@t-online.de (B.D.); heymut.omran@ukmuenster.de (H.O.); 2 Faculty of Medicine, University of Ljubljana, 1000 Ljubljana, Slovenia; marina.praprotnik@gmail.com; 3 Department for Pulmonary Diseases, University Children’s Hospital Ljubljana, University Medical Center Ljubljana, 1000 Ljubljana, Slovenia

**Keywords:** GRCh37, GRCh38, variant detection, primary ciliary dyskinesia, *ODAD4*, *TTC25*

## Abstract

Molecular diagnosis is highly sensitive to the human genome assembly used in a bioinformatics pipeline. Panels designed on GRCh37 (hg19) inherit its exon annotation, whereas transcript standards and interpretation databases are anchored to GRCh38 (hg38); a coding exon absent from GRCh37 can therefore be neither targeted nor analyzed. We describe two unrelated individuals with primary ciliary dyskinesia (PCD) in whom first-line, GRCh37-based panel sequencing was non-diagnostic. Both were re-analyzed on GRCh38 against the Matched Annotation from NCBI and EMBL-EBI (MANE) Select transcript. In Patient 1 (OP-880 II1), trio whole-genome sequencing identified a homozygous frameshift variant (NM_031421.5:c.1157del, p.(Lys386ArgfsTer52)) in exon 9 of *ODAD4* (previously *TTC25*), an exon not represented in GRCh37. In Patient 2 (OP-4121 II1), the panel detected only c.100C>T, p.(Gln34Ter); Sanger sequencing of exon 9 disclosed NM_031421.5:c.1273C>T, p.(Gln425Ter) in trans. Her cilia were immotile on high-speed video microscopy and lacked outer dynein arms on transmission electron microscopy. Two cases cannot establish frequency, but they identify a remediable failure mode: GRCh37-based pipelines miss GRCh38-defined exons, leaving a recessive genotype apparently monoallelic. Re-analysis on GRCh38 against MANE Select transcripts should be reflexive whenever a strong phenotype yields a negative or monoallelic result, including in previously tested individuals.

## 1. Introduction

Molecular diagnosis in genetic diseases rests on high-throughput DNA sequencing, in which short reads from targeted gene panels, whole-exome sequencing (WES) or whole-genome sequencing (WGS) are aligned to a single haploid human reference genome, and the resulting variants are called, filtered and interpreted according to established standards [1].

Two human genome reference builds remain in parallel clinical use: GRCh37, released in 2009 [2,3], and its successor GRCh38, released in 2013 [4]. These builds are produced by the Genome Reference Consortium (GRC) but are also commonly designated hg19 (referring to GRCh37) and hg38 (referring to GRCh38), following the naming convention of the University of California, Santa Cruz (UCSC) Genome Browser. The two designations are largely interchangeable, although minor sequence differences exist between corresponding GRC and UCSC releases [5,6]; for consistency, the GRC designations are used throughout this manuscript.

GRCh38 closed sequence gaps, corrected assembly errors and introduced alternate loci that improve read alignment and variant calling. Nevertheless, GRCh37 remains deeply embedded in diagnostic pipelines and annotation resources and is still widely used [4,7,8]. This persistence reflects the effort that a change in build demands: diagnostic pipelines were developed and validated on GRCh37, laboratory information systems hold legacy GRCh37 data, and variant annotations must be reconciled when moving to GRCh38-based transcripts [9]. The two builds differ not only in primary sequence but in the transcript annotations and the population and clinical variant databases anchored to them. The Matched Annotation from NCBI and EMBL-EBI (MANE) Select set, which defines one agreed clinical transcript per gene, is built on GRCh38 [10]. The Genome Aggregation Database (gnomAD), the principal population reference for allele frequencies, is likewise anchored to GRCh38 in its current releases, the latest of which aggregates more than 800,000 individuals [11,12,13]. Clinical variant interpretations are aggregated in ClinVar with genome-anchored coordinates [14]. Laboratories that remain on GRCh37 therefore operate with less complete transcript and population annotation, and GRCh37 carries a further, build-intrinsic liability: it stores the minor allele at roughly 1.9 million positions, so that individuals homozygous for the common allele are systematically miscalled [15,16].

These differences translate into discordant variant calls. In a systematic comparison of 1572 exomes, approximately 1.5% of single-nucleotide variants and 2.0% of insertions or deletions were called discordantly between GRCh37 and GRCh38 [8]. The discordant calls clustered in discrete problem regions, were enriched in a defined set of genes, several implicated in Mendelian disease, and arose predominantly from multi-mapped reads at duplicated or misassembled loci [8]. This discordance is not fully corrected by lifting variant coordinates from one build to the other because a proportion of variants cannot be lifted over and a further fraction change their predicted consequence after lift-over [8,17]. Conversely, realignment to the current build recovers variants that were absent on the older one, as shown by the re-analysis of tens of thousands of exomes in which many previously missing variants, including some in medically relevant genes, became detectable [18]. In genetic diseases, the consequence is immediate: if one of the two disease alleles lies in a region that the build or its annotation does not adequately represent, a genotype that is in truth homozygous or compound heterozygous is reported as non-diagnostic or monoallelic. Build-dependent loss of clinically relevant variants has been documented in individuals affected with other genetic disorders [19,20,21]. For example, in two siblings with developmental and epileptic encephalopathy, a pathogenic 13-bp duplication in *SYNGAP1* escaped detection by exome and high-depth targeted sequencing because the variant-bearing reads were either not aligned or were aligned to additional major histocompatibility complex contigs included in the hg19 reference [19]. In the opposite direction, variants in *U2AF1* were missed after the transition to GRCh38 because of changes in the GRCh38 reference sequence [20], and a false duplication in GRCh38 affects most of the coding region of *KCNE1* [21]. These examples concern heterozygous variants; in a recessive disorder, the same mechanism leaves one of the two required alleles unreported.

In this study, we use two unrelated individuals with primary ciliary dyskinesia (PCD), identified retrospectively in the PCD diagnostic cohort of the Department of General Pediatrics, University Hospital Münster, whose biallelic variants in *ODAD4*, previously named *TTC25*, were detected only after re-analysis on GRCh38, to show how this dependency operates in practice, and to define the reflex measures that recover a diagnosis when first-line analysis on GRCh37 returns a monoallelic or negative result. The two individuals were not ascertained to test this hypothesis; they are reported because, in both, the genetic diagnosis depended on the reference build.

PCD is a predominantly genetic motile ciliopathy caused by dysfunction of motile cilia, presenting with chronic upper and lower airway disease and, in about half of affected individuals, laterality defects [22,23]. Its definitive diagnosis increasingly depends on identifying biallelic variants in one of the numerous PCD-associated genes identified to date [22,23]. *ODAD4*, encoding the docking-complex protein ODAD4, is one such gene: biallelic loss-of-function variants cause a defect in outer dynein arm (ODA) assembly to the axonemes, resulting in PCD [24]. Because part of the *ODAD4* coding sequence lies in regions whose representation depends on the reference build and transcript used, *ODAD4* is well suited to expose how build choice affects recessive-variant detection.

## 2. Materials and Methods

### 2.1. Study Individuals and Ethical Approval

Two unrelated individuals with clinically suspected PCD (Patient 1, OP-880 II1; Patient 2, OP-4121 II1), evaluated according to the 2017 European Respiratory Society (ERS) diagnostic guideline [25], were studied; both also fulfilled the diagnostic criteria of the current joint ERS/American Thoracic Society (ATS) guideline [26]. Written informed consent was obtained from the participants or their legal guardians, and the study was approved by the ethics committee of the University of Münster on 2 March 2015 and 29 March 2016 (Az. 2015-104-f-S, 2016-136-f-S). Both individuals were identified retrospectively in the PCD diagnostic cohort of the Department of General Pediatrics, University Hospital Münster. Nasal brushings from healthy volunteers without respiratory disease, obtained under the same ethics approval, served as controls for high-speed video microscopy, transmission electron microscopy and immunofluorescence. For Patient 1 (OP-880 II1), only externally referred genomic DNA was available, which restricted analysis to sequencing; Patient 2 (OP-4121 II1) underwent the complete diagnostic work-up in addition to genetic testing.

### 2.2. Targeted Gene-Panel Sequencing on GRCh37

Genomic DNA was extracted from peripheral blood using standard procedures [27]. Targeted next-generation sequencing was performed as part of the routine diagnostic work-up, using different capture panels and sequencing platforms because the analyses were conducted several years apart.

For Patient 1 (OP-880 II1), genomic DNA was fragmented, and coding exons with approximately 20 bp of flanking intronic sequence were enriched using Illumina TruSight Rapid Capture technology and sequenced on an Illumina MiSeq platform (Illumina, San Diego, CA, USA). Mean sequencing depth exceeded 250×, with a minimum required coverage of 30×.

For Patient 2 (OP-4121 II1), analyzed approximately three years later, target enrichment was performed using a customized Twist hybridization-capture panel (Twist Bioscience Corporation, South San Francisco, CA, USA), followed by sequencing on an Illumina NextSeq 1000 platform.

Both panels covered all PCD-associated genes established at the respective time of analysis, including *ODAD4* (Supplementary Table S2), and were designed and analyzed against the GRCh37 reference genome. Sequence data were processed using SeqPilot software V. 5.1.0 (JSI Medical Systems GmbH, Ettenheim, Germany). Variants were evaluated using the population databases available at the time, including the 1000 Genomes Project, dbSNP, ExAC and an in-house database, and classified according to the recommendations of the American College of Medical Genetics and Genomics and the Association for Molecular Pathology (ACMG/AMP) [1]. Variant nomenclature followed Human Genome Variation Society (HGVS) recommendations.

### 2.3. Whole-Genome Sequencing on GRCh38

After targeted panel sequencing had returned no causative variant in Patient 1 (OP-880 II1), trio whole-genome sequencing (WGS) of the index individual and both parents was performed at the Cologne Center for Genomics (CCG), University of Cologne. Libraries were prepared and size-selected using the CCG in-house polymerase chain reaction (PCR)-free protocol based on the Illumina TruSeq DNA Sample Preparation Kit (Illumina, San Diego, CA, USA) and Agencourt AMPure XP beads (Beckman Coulter, Brea, CA, USA), starting from 1.2 μg of genomic DNA, followed by a single PCR cycle only to complete the adapter structure. Libraries were validated on an Agilent 2200 TapeStation (Agilent Technologies, Santa Clara, CA, USA) and quantified. Sequencing was performed on an Illumina NovaSeq 6000 platform in paired-end mode. Paired-end reads were aligned to the GRCh38 human reference genome. Duplicate reads were marked with Picard (https://broadinstitute.github.io/picard/; first accessed 3 May 2021, last accessed on 8 September 2026), and base quality score recalibration and local indel realignment were performed with GATK [28]. Alignment and coverage statistics were computed with Picard on the resulting BAM files. Variants were called genome-wide and annotated with software developed at the CCG on the basis of Ensembl gene models. Low-confidence calls were excluded (alternative allele frequency < 10%, coverage at the variant position < 5 reads, variant quality score < 10, fewer than 3 variant-supporting reads). Remaining variants were annotated for their presence in dbSNP, gnomAD v4, ClinVar, dbVar and DGVa, for GERP conservation scores, and against the commercial HGMD Professional database and an in-house CCG exome collection. Effects on splicing were predicted using the maximum entropy approach of Yeo and Burge [29], and SIFT [30], PolyPhen-2 [31] and RVIS [32] scores were considered for all coding variants. Runs of homozygosity were computed from the genome-wide variant list using Allegro [33]. Annotated variant lists were uploaded to the CCG Varbank platform (https://varbank.ccg.uni-koeln.de; accessed on 17 September 2021) and Varbank 2 (https://varbank.ccg.uni-koeln.de/varbank2/; accessed on 17 September 2021 and following) for filtering and evaluation. Candidate variants were prioritized under an autosomal-recessive model, assessed against the MANE Select transcript of *ODAD4* (NM_031421.5), classified according to the ACMG/AMP criteria [1], and confirmed together with parental segregation by Sanger sequencing.

### 2.4. Variant Filtering, Interpretation and Nomenclature

Variants were filtered under an autosomal-recessive model, retaining rare, protein-altering and canonical splice-site alleles and excluding those with a minor-allele frequency of 0.01 or greater in gnomAD [11,12]. Candidate variants were checked against ClinVar for prior clinical assertions [14], classified according to the ACMG/AMP criteria [1], and described following HGVS nomenclature relative to NM_031421.5 [34]. Only variants classified as pathogenic (class 5) or likely pathogenic (class 4) were considered disease-causing.

### 2.5. Transcript Re-Annotation and Exon-Directed Sanger Sequencing

In Patient 2 (OP-4121 II1), in whom targeted gene-panel sequencing on GRCh37 identified only a single heterozygous variant, the second variant was in exon 9 of *ODAD4*, which is absent from the GRCh37 assembly. This exon can therefore be neither targeted in a GRCh37-based panel design nor correctly aligned or annotated in a GRCh37-based analysis, irrespective of the transcript used. The MANE Select *ODAD4* reference transcript NM_031421.5 (12 coding exons) was therefore mapped against GRCh37 and GRCh38 to identify the missing region [10]. Gene-specific primers flanking exon 9 were designed on the GRCh38 sequence (Supplementary Table S1). Exon 9 was amplified by PCR and sequenced bidirectionally on a 3730xl DNA Analyzer (Applied Biosystems, Thermo Fisher Scientific, Waltham, MA, USA); trace files were aligned to NM_031421.5 and analyzed with CodonCode Aligner (version 3.7.1; CodonCode Corporation, Centerville, MA, USA). The same assay, together with an equivalent assay for exon 1, was applied to the available family members to establish allelic phase and segregation.

### 2.6. Functional and Ultrastructural Assessment

In Patient 2 (OP-4121 II1), ciliary function and ultrastructure were assessed to corroborate pathogenicity of the identified variants. Nasal nitric oxide (nNO), which is markedly reduced in most individuals with PCD and is therefore used as a diagnostic test, was measured according to standardized recommendations [25,35,36]. Ciliary beating was evaluated by high-speed video microscopy analysis (HSVMA) on native and air–liquid interface (ALI)-cultured respiratory epithelial cells. Axonemal ultrastructure was examined by transmission electron microscopy (TEM) and classified according to the international BEAT-PCD consensus criteria [37]. The axonemal localization of the ODA heavy chain DNAH5 and of ODAD4 was assessed by immunofluorescence (IF) microscopy in comparison with healthy control cells [38].

### 2.7. Respiratory Epithelial Cells and Air–Liquid Interface Culture

Respiratory epithelial cells were obtained from the middle turbinate by nasal brush biopsy (Engelbrecht Medizin- und Labortechnik, Germany) and suspended in RPMI medium supplemented with antibiotic-antimycotic (both Gibco, Thermo Fisher Scientific, Waltham, MA, USA). The cell suspension was used directly for HSVMA, TEM and IF, or for ALI culture. For ALI culture, cells were pre-cultured in rat collagen-coated flasks, detached by collagenase and trypsin treatment, expanded in PneumaCult-Ex or PneumaCult-Ex Plus medium and differentiated in PneumaCult-ALI medium (STEMCELL Technologies, Vancouver, BC, Canada), as described previously [39,40]. Differentiated cultures were used for HSVMA and IF.

### 2.8. High-Speed Video Microscopy

Ciliary beat frequency and beat pattern were assessed by HSVMA on freshly brushed nasal respiratory epithelial cells and, after differentiation, on ALI cultures. Freshly brushed cells were examined directly. ALI-cultured cells were detached with 0.05% trypsin-EDTA at 37 °C; the reaction was stopped with heat-inactivated fetal bovine serum (both Gibco), and the cells were resuspended in RPMI medium with antibiotic-antimycotic and spread onto a glass slide. Videos were recorded at 37 °C with a Basler acA1300-200 μm monochrome high-speed camera (Basler, Ahrensburg, Germany) attached to an inverted phase-contrast microscope (Axio Vert.A1; Zeiss, Oberkochen, Germany) equipped with a 40× objective and were analyzed with the Sisson-Ammons Video Analysis (SAVA) software version 2.1.2.W [41]. Beating was scored by experienced observers as normal, stiff, dyskinetic, or immotile, according to established diagnostic criteria and following our time- and temperature-controlled protocols [42,43,44].

### 2.9. Transmission Electron Microscopy

Respiratory epithelial cells obtained by nasal brush biopsy were fixed in 2.5% glutaraldehyde and post-fixed in 1% osmium tetroxide. Samples were infiltrated overnight at 4 °C with a 1:1 mixture of 1,2-epoxypropane and Epon before embedding. Ultrathin sections were transferred onto copper grids, contrasted with uranyl acetate and Reynolds’ lead citrate, and examined with a Philips CM10 transmission electron microscope (Philips, Eindhoven, The Netherlands), as described previously [45]. A total of 50 axonemal cross-sections from different ciliated cells of Patient 2 (OP-4121 II1) were assessed systematically, in comparison with control samples, for the presence or absence of the ODAs and for the integrity of the central pair, radial spokes and nexin-dynein regulatory complex (N-DRC), and were classified according to the international BEAT-PCD consensus criteria for reporting TEM in PCD [37].

### 2.10. Immunofluorescence

ALI-differentiated respiratory cells were spread onto glass slides and air-dried, fixed in 4% paraformaldehyde for 15 min at room temperature, permeabilized with Triton X-100 for 10 min, blocked in 1% skim milk for 3 h, and double-labeled overnight at 4 °C with primary antibodies directed against the ODA heavy chain DNAH5 (mouse monoclonal, self-generated), the N-DRC component GAS8 (rabbit polyclonal 1:300; HPA041311; Atlas Antibodies (Bromma, Sweden)) and against ODAD4 (rabbit polyclonal 1:300; HPA023908; Atlas Antibodies), together with an axonemal marker antibody (anti-acetylated α-tubulin; mouse monoclonal 1:10,000; T6793; Sigma-Aldrich (St. Louis, MO, USA)). After incubation with fluorophore-conjugated secondary antibodies (goat anti-mouse Alexa Fluor 488, A11029, and goat anti-rabbit Alexa Fluor 546, A11035; Invitrogen (Carlsbad, CA, USA); 1:1000) for 30 min at room temperature, DNA was stained with Hoechst 33342 (Sigma-Aldrich). Images were taken with a Zeiss Apotome Axiovert 200 (Carl Zeiss Microscopy GmbH, Jena, Germany) using a 60× objective and processed with AxioVision 4.8, ZEN and Adobe Creative Suite 4 software. The axonemal localization of DNAH5 and ODAD4 was compared with that in respiratory cells from healthy control individuals to detect absence or mislocalization of ODA and docking-complex components, following our established immunofluorescence protocols [24,38,46].

## 3. Results

### 3.1. Patient 1 (OP-880 II1): A Homozygous ODAD4 Variant Detected Only on GRCh38

**Figure 1:**
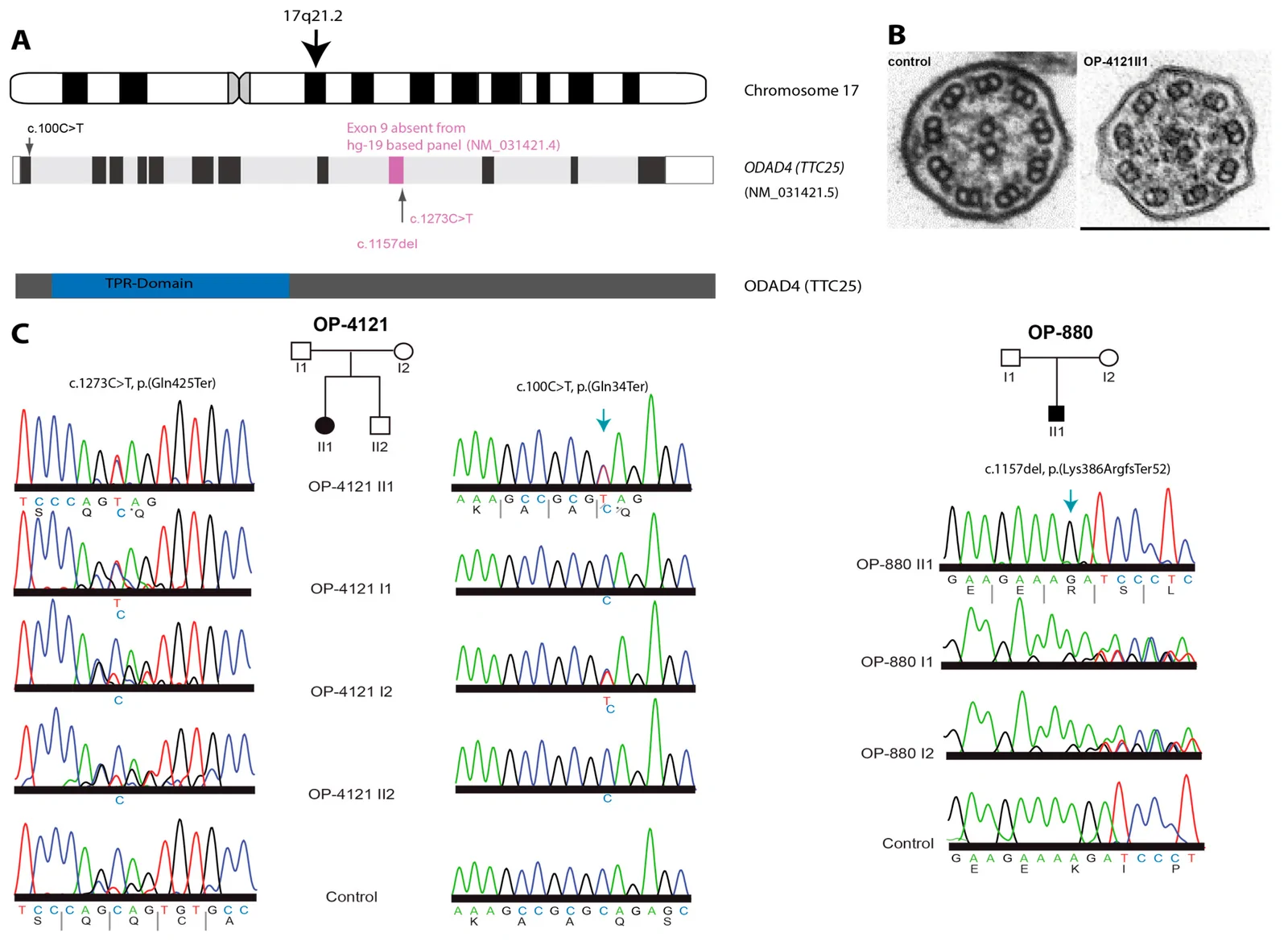
Chromosomal localization, transcript structure, ultrastructure and segregation of *ODAD4* variants in two unrelated individuals with PCD. (**A**) Schematic of chromosome 17 with the arrow marking the *ODAD4* locus at 17q21.2. Below, the exon–intron structure of the MANE Select reference transcript NM_031421.5 is shown (filled black boxes: coding exons; filled gray boxes: introns; open boxes: 5′ and 3′ untranslated regions). The three truncating variants identified in this study are indicated by arrows: c.100C>T, p.(Gln34Ter) and c.1273C>T, p.(Gln425Ter) in Patient 2 (OP-4121 II1), and c.1157del, p.(Lys386ArgfsTer52) in Patient 1 (OP-880 II1). The exon highlighted in magenta (exon 9 of NM_031421.5) is not represented in the GRCh37 assembly and was therefore not interrogated by first-line targeted sequencing. However, it harbors two of the three pathogenic alleles. Below is a schematic of the 672-residue ODAD4 protein drawn to scale, with the tetratricopeptide repeat (TPR) domain in blue. All three variants are predicted to truncate the protein. (**B**) Transmission electron microscopy (TEM) of respiratory epithelial cross-sections from a healthy control (left) and from Patient 2 (OP-4121 II1) (right). The control axoneme shows the canonical 9 × 2 + 2 configuration with outer and inner dynein arms. In Patient 2, the 9 × 2 + 2 configuration, central pair, and radial spokes are preserved, whereas the outer dynein arms are absent from all peripheral doublets. Scale bar, 200 nm. (**C**) Pedigrees of the families of Patient 2 (OP-4121 II1) (left) and Patient 1 (OP-880 II1) (right). Representative electropherograms are shown for each family member analyzed and for a healthy control; arrows indicate the variant positions, with the corresponding nucleotide and amino acid sequences given below each trace. In the family of Patient 2 (OP-4121 II1), the index individual (II1) carries c.1273C>T, p.(Gln425Ter), inherited from the father (I1), and c.100C>T, p.(Gln34Ter), inherited from the mother (I2), confirming compound heterozygosity; the unaffected sibling (II2) carries neither variant. Mild background signal at positions adjacent to c.1273 in the trace of II2 did not affect genotyping (see Section 3). In the family of Patient 1 (OP-880 II1), the index individual (II1) is homozygous for c.1157del, p.(Lys386ArgfsTer52), and both parents (I1, I2) are heterozygous carriers. Variants are annotated according to Human Genome Variation Society nomenclature relative to NM_031421.5. * denotes a stop codon.

Patient 1 (OP-880 II1), a 19-year-old man, had a history of chronic rhinosinusitis and recurrent otitis media together with situs inversus totalis. This combination of oto-sino-pulmonary disease and *situs inversus totalis* corresponds to the Kartagener phenotype and meets the clinical criteria that establish a strong suspicion of PCD [25,26]. First-line targeted panel sequencing, aligned to the GRCh37 reference and covering *ODAD4* among the analyzed PCD genes, returned no causative variant. Because the clinical suspicion remained high, trio-WGS was performed with alignment to the GRCh38 reference and identified a homozygous frameshift variant in *ODAD4* (NM_031421.5:c.1157del, p.(Lys386ArgfsTer52)) (Figure 1). The deletion was found to lie in exon 9 and shifts the reading frame, introducing a premature termination codon (PTC) after 51 aberrant residues. This predicts a truncated protein of 436 of the 672 residues of ODAD4. The deletion is carried by both parents in a heterozygous state. The variant is absent from gnomAD [11,12], had not been reported previously, and met the ACMG/AMP criteria for a (likely) pathogenic variant (PVS1, PM2) [1]. Owing to the external origin and the limited quantity of the referred specimen, HSVMA, ciliary transport analysis, TEM and IF could not be performed for this individual. Nonetheless, the homozygous truncating *ODAD4* variant, in the context of a classic clinical phenotype, established a molecular diagnosis of *ODAD4*-related PCD (Table 1).

### 3.2. Patient 2 (OP-4121 II1): Compound Heterozygosity Revealed Only After MANE-Based Re-Annotation

Patient 2 (OP-4121 II1), a 9-year-old girl born to non-consanguineous parents, presented with chronic respiratory symptoms. Her nNO level was markedly reduced at 5.8 ppb, corresponding to a production rate of 2.2 nL/min. This value lies far below the standardized disease-specific cut-off of 77 nL/min and is therefore strongly supportive of PCD [35,36]. First-line panel sequencing on the GRCh37 reference detected only a single heterozygous nonsense variant in *ODAD4* (NM_031421.5:c.100C>T, p.(Gln34Ter)) in exon 1. Targeted Sanger sequencing across the family showed that this allele was carried by the proband and her mother but not by the father or the brother (II2); a single heterozygous pathogenic allele could not, by itself, explain a recessive genetic disorder. Re-examination of the *ODAD4* reference transcript NM_031421.5 on GRCh38 showed that the coding sequence spans 12 exons, whereas the corresponding region on GRCh37 captured only 11. Exon 9 could not be resolved, reflecting a gap in the GRCh37 assembly and leaving this coding exon unexamined. Exon-specific amplification and bidirectional Sanger sequencing of this previously unexamined exon 9 disclosed a second nonsense variant (NM_031421.5:c.1273C>T, p.(Gln425Ter)) (Figure 1). Family testing confirmed that this variant was present in the proband and her father but absent in her mother and brother, consistent with the two nonsense variants lying on opposite alleles and the proband being compound heterozygous.

The electropherogram of the unaffected brother (II2) showed mild background signal at positions adjacent to c.1273 but was concordant with the segregation pattern (Figure 1C). Read as sequence changes, these low-level secondary peaks would correspond to NM_031421.5:c.1272G>C, p.(Gln424His), c.1274A>C, p.(Gln425Pro), and c.1275G>T, p.(Gln425His), which are classified as likely benign, of uncertain significance and likely benign, respectively. They did not affect genotyping at c.1273 in any family member and were not considered further.

Both variants were absent from gnomAD [11,12]. The c.1273C>T variant had not been reported previously, whereas c.100C>T has been described in the compound heterozygous state in an individual with PCD [47]. Each satisfied the ACMG/AMP criteria for a (likely) pathogenic variant (PVS1, PM2), further supported by their confirmed segregation in trans (PM3) (Table 1) [1].

### 3.3. Ciliary Phenotype Corroborates Loss of Function in Patient 2 (OP-4121 II1)

**Figure 2:**
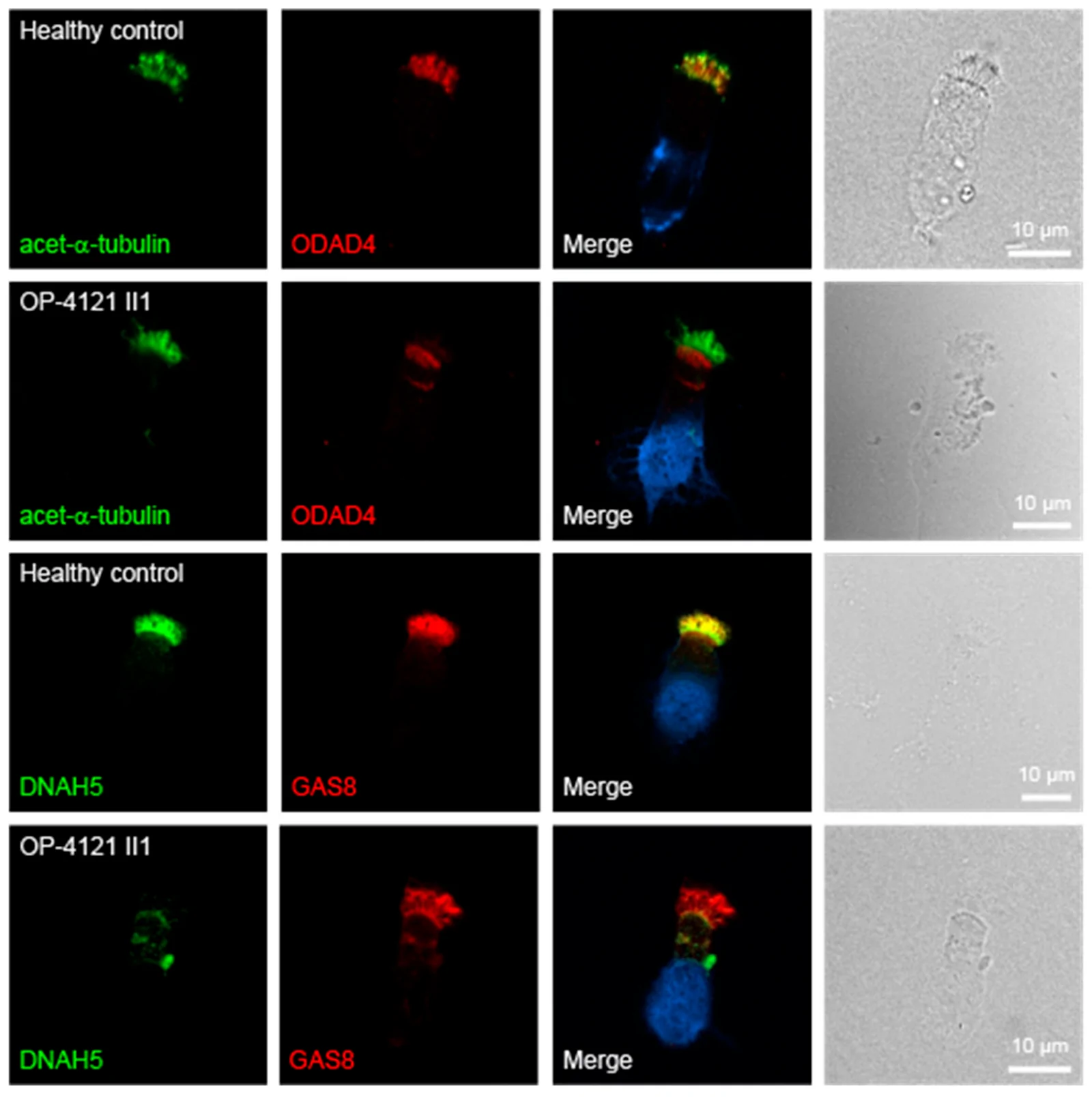
ODAD4 and DNAH5 are absent from the ciliary axonemes of respiratory epithelial cells from an individual with biallelic pathogenic variants in *ODAD4*. ALI-differentiated respiratory epithelial cells from a healthy control and from Patient 2 (OP-4121 II1), who carries biallelic *ODAD4* variants, are double-labeled with antibodies directed against acetylated α-tubulin (green) and ODAD4 (red), or against DNAH5 (green) and GAS8 (red). Nuclei are stained with Hoechst 33342 (blue). In unaffected controls, ODAD4 and DNAH5 localize along the entire axonemal length. In cells from Patient 2 (OP-4121 II1), both proteins are absent from the axonemes, whereas GAS8 localizes normally along the axonemes. These findings are consistent with the pathogenic *ODAD4* variants. Scale bars, 10 µm. Right column, differential interference contrast images of the same cells.

The availability of a respiratory epithelial sample enabled Patient 2 (OP-4121 II1) to undergo the complete functional and structural work-up. On HSVMA, the cilia were essentially immotile, with only occasional residual twitching and no measurable beat frequency or coordinated waveform. The same pattern was present in freshly brushed cells and in ALI-differentiated cultures, indicating a fixed, intrinsic motility defect rather than a secondary or acquired abnormality (Supplementary Videos S1 and S2). TEM of axonemal cross-sections demonstrated an intact 9 × 2 + 2 configuration, with a normal central pair, radial spokes and N-DRCs, but a complete absence of the ODAs from all 50 cross-sections examined (Figure 1B), a hallmark ultrastructural defect in PCD [37] and the expected consequence of a defective ODA-docking machinery [24]. IF analysis showed that ODAD4 and the ODA heavy chain DNAH5 were absent from the ciliary axonemes of Patient 2 (OP-4121 II1), whereas both localized along the entire axonemal length in control cells (Figure 2). The residual signal at the ciliary base and in the cytoplasm reflects non-specific background staining by the rabbit polyclonal antibody. The N-DRC component GAS8 localized normally along the axonemes, confirming that the cilia were present and that the defect was confined to the ODA compartment (Figure 2). Both nonsense alleles are predicted to cause loss of function through nonsense-mediated mRNA decay or truncation of the protein. ODAD4 was thus not incorporated into the axoneme, and the concomitant axonemal loss of DNAH5 links the docking-complex defect to a failure of ODA assembly, consistent with the outer arms missing on TEM [24]. Taken together, the genetic, functional and ultrastructural findings were mutually corroborating and established *ODAD4*-related PCD with complete loss of the ODAs. The presence of a hallmark ODA defect on TEM, together with the markedly reduced nNO, the immotile ciliary phenotype and the biallelic *ODAD4* variants, fulfills the criteria for a definitive diagnosis of PCD in Patient 2 (OP-4121 II1) according to the 2017 ERS guideline [25] and for confirmed PCD according to the current joint ERS/ATS guideline [26].

## 4. Discussion

### 4.1. Two Individuals in Whom the Reference Build Determined the Diagnosis

That first-line testing failed to resolve both cases, despite an established disease gene, clear truncating alleles and adequate sequencing, points to the reference build and transcript annotation, not the assay, as the cause.

At the level of a single gene, this makes concrete a property of the two reference builds that has been quantified across large cohorts. The reference build and transcript used for mapping and annotation, not the sequencing chemistry or assay design, determined the diagnostic outcome. In each individual, the disease allele that first-line testing did not report lay in a coding region that is absent from the GRCh37 reference used for read alignment and variant calling yet fully represented in the GRCh38-anchored MANE Select transcript.

In Patient 1 (OP-880 II1), first-line testing yielded a non-diagnostic result that was resolved only by WGS analyzed on GRCh38. In Patient 2 (OP-4121 II1), it yielded an apparently monoallelic result that was resolved into a biallelic genotype only by exon-directed re-sequencing guided by the MANE Select transcript. Re-evaluation against GRCh38 and MANE Select thus converted a non-diagnostic or monoallelic result into a confirmed biallelic, disease-causing genotype in each individual.

This is consistent with the systematic difference between the two builds. Relative to GRCh37, GRCh38 corrected assembly errors, closed gaps, and added alternate loci, which improves read alignment and variant calling [4,7]. Nevertheless, across 1572 exomes, about 1.5% of single-nucleotide variants and 2.0% of insertions or deletions were called discordantly between the builds [8]. These discordant calls cluster in discrete problem regions and are enriched in a defined set of genes, several of them linked to Mendelian disease, and they arise predominantly from multi-mapped reads at duplicated or misassembled loci [8]. Curated Genome in a Bottle (GIAB) benchmarks for medically relevant genes, together with the build-specific failures described in the Introduction, show that some clinically important loci remain vulnerable to the choice of reference build [20,48]. Independent of these regional effects, GRCh37 carries the minor allele at roughly 1.9 million positions, so that individuals homozygous for the common allele are systematically miscalled, a source of both false-negative and false-positive results that is intrinsic to the older assembly [15,16]. A recessive genotype is especially vulnerable to such effects: because a diagnosis requires both alleles, failure to represent a single exon or locus is sufficient to leave the case unsolved, exactly as observed here.

### 4.2. Migration Is Not Fully Substituted by Coordinate Lift-Over

A common interim strategy is to keep GRCh37-based pipelines and lift variant coordinates over to GRCh38, but this does not fully reproduce the benefit of native GRCh38 analysis. Some variants cannot be lifted over, and others change their predicted molecular consequence after conversion, so lift-over neither recovers all build-specific calls nor guarantees stable interpretation [8,17]. In the present cases, lift-over could not have recovered the missing alleles, since exon 9 is absent from GRCh37 and no variant was called there. A laboratory that adopts lift-over as an interim solution therefore cannot assume that previously reported negative or monoallelic results have been re-examined. Conversely, realignment to GRCh38 recovers variants absent on GRCh37, including in medically relevant genes, as shown by the re-analysis of tens of thousands of exomes [18]. For a laboratory, this means that a variant missing on GRCh37 may be present on GRCh38 not because it was called more leniently, but because the newer assembly represents its locus correctly.

### 4.3. Build-Anchored Databases Compound the Effect

The reference build also determines which annotation and reference resources are available for interpretation. The MANE Select set, which defines one agreed clinical transcript per gene and thereby standardizes which exons are considered coding, is anchored to GRCh38. In Patient 2 (OP-4121 II1), it was the MANE Select transcript that revealed the coding exon missing from GRCh37 and directed the re-sequencing that established compound heterozygosity [10]. The main population reference, gnomAD, is likewise anchored to GRCh38 in its current release, which aggregates more than 800,000 individuals and provides substantially broader allele-frequency data than earlier, GRCh37-based versions [11,12,13]. Clinical interpretations aggregated in ClinVar are used within pipelines and annotation tools that are themselves migrating to GRCh38 [9,14]. Laboratories that remain on GRCh37 therefore work not only with a less accurate assembly but with less complete transcript, frequency and clinical annotation, all of which influence whether a rare recessive allele is detected, retained through filtering and correctly classified.

### 4.4. Practical Measures

The corrective measures follow directly from these findings and are consistent with current laboratory guidance for the transition from GRCh37 to GRCh38 [9]. They comprise (i) migration of alignment and variant calling to GRCh38; (ii) adoption of MANE Select and MANE Plus Clinical transcripts for coverage design and reporting; (iii) use of GRCh38-anchored population and clinical databases; (iv) alt-aware alignment and masking of the falsely duplicated regions in GRCh38; (v) at the case level, treating an apparently monoallelic result in an individual with a strong clinical suspicion of a recessive disease not as exclusionary but as a trigger for reflex re-annotation, exon-directed re-sequencing, or exome or genome sequencing; and (vi) at the laboratory level, systematic re-analysis on GRCh38 of previously tested individuals whose results were negative or monoallelic despite a strong clinical suspicion of a recessive disorder, with issue of updated reports [9]. In the present cases, native GRCh38 analysis and MANE-guided exon-directed re-sequencing were decisive: the biallelic *ODAD4* genotype, and with it the molecular diagnosis of *ODAD4*-related PCD, was established only after the analysis moved to GRCh38 and the MANE Select transcript.

Beyond GRCh38, more complete references such as the telomere-to-telomere CHM13 assembly (T2T-CHM13) and the human pangenome reference aim to represent loci that remain unresolved or misassembled in the current build [49,50]. T2T-CHM13 resolves the remaining gaps of GRCh38, including centromeric and segmentally duplicated regions, and improves short-read variant calling in many difficult loci [21,49]. However, its clinical use is currently limited because MANE, gnomAD and ClinVar remain anchored to GRCh38. Long-read sequencing complements these references: by spanning duplicated and repetitive loci that generate multi-mapped short reads, it directly addresses a principal mechanism of build-dependent discordance [8,51].

## 5. Conclusions

The problem illustrated here is transitional but not yet resolved. As diagnostic laboratories complete the migration to GRCh38, the residual gap will shift to loci that even GRCh38 represents imperfectly. Until more complete references and long-read sequencing are routine in clinical practice, the practical priorities for recessive-disease diagnostics are clear: analyze on GRCh38, design and report against MANE Select transcripts, interpret against GRCh38-anchored databases, and re-examine monoallelic results before excluding a gene. Because lift-over of existing GRCh37 calls cannot recover alleles that were never called, migration should be accompanied by a re-analysis of archived cases that remained negative or monoallelic. *ODAD4* in PCD illustrates this, but the principle applies to other Mendelian disorders as well.

## Figures and Tables

**Table 1:** Genetic findings.

Patient (Sex, Age)	cDNA (NM_031421.5)	Protein	Zygosity	Parental Origin	gnomAD	ACMG/AMP
Patient 1, OP-880 II1 (M, 19)	c.1157del	p.(Lys386ArgfsTer52)	Homozygous	both	Absent	(Likely) pathogenic (PVS1, PM2)
Patient 2, OP-4121 II1 (F, 9)	c.100C>T	p.(Gln34Ter)	Heterozygous	Maternal	Absent	(Likely) pathogenic (PVS1, PM2, PM3)
Patient 2, OP-4121 II1 (F, 9)	c.1273C>T	p.(Gln425Ter)	Heterozygous	Paternal	Absent	(Likely) pathogenic (PVS1, PM2, PM3)

## Data Availability

All data supporting this study are available in the main text and on request from the corresponding author. Primer sequences for Sanger sequencing are provided in Supplementary Table S1.

## Supplementary Materials

The following supporting information can be downloaded at: https://www.mdpi.com/article/10.3390/cells15201893/s1, Table S1: Primer sequences for PCR amplification and Sanger sequencing of *ODAD4* (*TTC25*) exons 1 and 9 (NM_031421.5). Primers were designed on the GRCh38 reference sequence; amplicon coordinates were obtained by matching the primer sequences to the GRCh38 reference. The exon 1 amplicon spans coding exon 1 (c.1–c.114, including c.100), and the exon 9 amplicon spans coding exon 9 (c.1146–c.1342, including c.1157 and c.1273) of NM_031421.5. The exon 9 primers have no binding site in the GRCh37 *ODAD4* locus. Table S2: Genes covered by the targeted PCD gene panels (n = 67) and the RefSeq transcripts used for variant analysis; Video S1: High-speed video microscopy of respiratory cilia from a healthy control; Video S2: High-speed video microscopy of respiratory cilia from Patient 2 (OP-4121 II1) (ALI-differentiated cells).
