# Supplementary material for: Re-Analysis on GRCh38 with the MANE Select Transcript Resolves Previously Undetected Biallelic ODAD4 Variants in Primary Ciliary Dyskinesia
Source: Cells. 2026 Oct 17;15(20):1893. doi: 10.3390/cells15201893 (PMC13703574; doi:10.3390/cells15201893)
Supplement: Supplementary file 1 [file cells-15-01893-s001.zip › Supplementary_Materials_cells-4619957.pdf]

Supplementary Materials

Re-analysis on GRCh38 with the MANE Select transcript resolves previously undetected biallelic *ODAD4* variants in primary ciliary dyskinesia

Petra Pennekamp, Marina Praprotnik, Bernd Dworniczak, Heymut Omran and Rim Hjeij

Table S1. Primer sequences for PCR amplification and Sanger sequencing of *ODAD4* (*TTC25*) exons 1 and 9 (NM_031421.5).

Primer	Sequence (5′→3′)	Product size	Amplicon (GRCh38)
TTC25_ex1F	gcaacagcaaacactaaccag	403 bp	chr17:41,930,472–41,930,874
TTC25_ex1R	gtgatggggtgatggatagg		
TTC25_NEW_ex9F_3	gttttattccgccacccagc	507 bp	chr17:41,949,058–41,949,564
TTC25_NEW_ex9R_3	aggtaggctcaaggtacccc		

Primers were designed on the GRCh38 reference sequence; amplicon coordinates were obtained by matching the primer sequences to the GRCh38 reference. The exon 1 amplicon spans coding exon 1 (c.1–c.114, including c.100), and the exon 9 amplicon spans coding exon 9 (c.1146–c.1342, including c.1157 and c.1273) of NM_031421.5. The exon 9 primers have no binding site in the GRCh37 *ODAD4* locus.

Table S2. Genes covered by the targeted PCD gene panels (n = 67) and the RefSeq transcripts used for variant analysis.

Gene	RefSeq transcript(s)	Gene	RefSeq transcript(s)
AK8	NM_152572.3	*DNAI2*	NM_001353167.2
ARMC4/ODAD2	NM_018076.5	*DNAJB13*	NM_153614.4
CBY1	NM_015373.4	*DNAL1*	NM_031427.4, NM_001201366.2
CCDC39	NM_181426.2	*DRC1*	NM_145038.5
CCDC40	NM_017950.4, NM_001330508.2, NM_001243342.2	*DYNC2H1*	NM_001080463.2
CCDC65/DRC2	NM_033124.5	*ENKUR*	NM_145010.4, NM_001270383.2
CCDC103/DNAAF19	NM_213607.3	*FOXJ1*	NM_001454.4
CCDC114/ODAD1	NM_001364171.2, NM_144577.4	*GAS2L2*	NM_139285.4
CCDC151/ODAD3	NM_145045.5	*GAS8/DRC4*	NM_001481.3
CCNO	NM_021147.5	*HYDIN*	NM_001270974.2
CFAP45	NM_012337.3	*IFT74*	NM_025103.4
CFAP46	NM_001200049.3	*KIAA0586/TALPID3*	NM_001329943.3
CFAP52	NM_145054.5	*LRRC56/DNAAF12*	NM_198075.4
CFAP53	NM_145020.5	*MCIDAS*	NM_001190787.3
CFAP54	NM_001306084.2	*MNS1*	NM_018365.4
CFAP74	NM_001304360.2	*NEK10*	NM_001394966.1, NM_001394970.1
CFAP221	NM_001271049.2	*NME5/RSPH23*	NM_003551.3
CFAP298/DNAAF16	NM_021254.4	*OFD1*	NM_003611.3, NM_001330210.2
CFAP300/DNAAF17	NM_032930.3	*RPGR*	NM_000328.3
CLXN/ODAD5	NM_024593.4, NM_001142857.2	*RSPH1*	NM_080860.4
DAW1/DNAAF18	NM_178821.3, NM_001330004.2	*RSPH3*	NM_031924.8
DNAAF1	NM_178452.6, NM_001318756.1	*RSPH4A*	NM_001010892.3

Gene	RefSeq transcript(s)	Gene	RefSeq transcript(s)
DNAAF2	NM_018139.3	*RSPH9*	NM_001193341.2
DNAAF3	NM_001256714.1	*SPAG1/DNAAF13*	NM_003114.5, NM_172218.3
DNAAF4	NM_130810.4, NM_001033560.2	*SPEF2*	NM_024867.4
DNAAF5	NM_017802.4	*STK36*	NM_015690.5
DNAAF6/PIH1D3	NM_001169154.2	*TP73*	NM_005427.4, NM_001126242.3, NM_001204192.2
DNAAF11/LRRC6	NM_012472.6, NM_001321965.2	*TTC12*	NM_001318533.2, NM_001378064.1, NM_001378063.1
DNAH1	NM_015512.5	*TTC25/ODAD4*	NM_031421.5
DNAH5	NM_001369.3	*TUBB4B*	NM_006088.6
DNAH9	NM_001372.4	*TXNDC3/NME8*	NM_016616.5
DNAH10	NM_001372106.1	*WDR65/CFAP57*	NM_001378189.1
DNAH11	NM_001277115.2	*ZMYND10/DNAAF7*	NM_015896.4
DNAI1	NM_001281428.2		

Where two gene symbols are given, they denote the same gene under its current and a previous or alternative symbol.

Video S1. High-speed video microscopy of respiratory cilia from a healthy control.

Video S2. High-speed video microscopy of respiratory cilia from Patient 2 (OP-4121 II1) (ALI-differentiated cells).
